# First person – Ming-Hsuan Wen

**DOI:** 10.1242/bio.060260

**Published:** 2024-03-12

## Abstract

First Person is a series of interviews with the first authors of a selection of papers published in Biology Open, helping researchers promote themselves alongside their papers. Ming-Hsuan Wen is first author on ‘ Deterministic nuclear reprogramming of mammalian nuclei to a totipotency-like state by Amphibian meiotic oocytes for stem cell therapy in humans’, published in BiO. Ming-Hsuan conducted the research described in this article while a PhD student and postdoc in Prof. John Gurdon's lab at Wellcome/Cancer Research UK Gurdon Institute, Cambridge, UK. She is now a Founder and CEO of NUWA Therapeutics, spun-out of University of Cambridge and a visiting researcher at the University of Cambridge in the lab of Prof. John Gurdon at Wellcome/Cancer Research UK Gurdon Institute, Cambridge, UK, investigating advancing the cellular reprogramming technology and providing safe and high-performing stem cells for novel therapies.


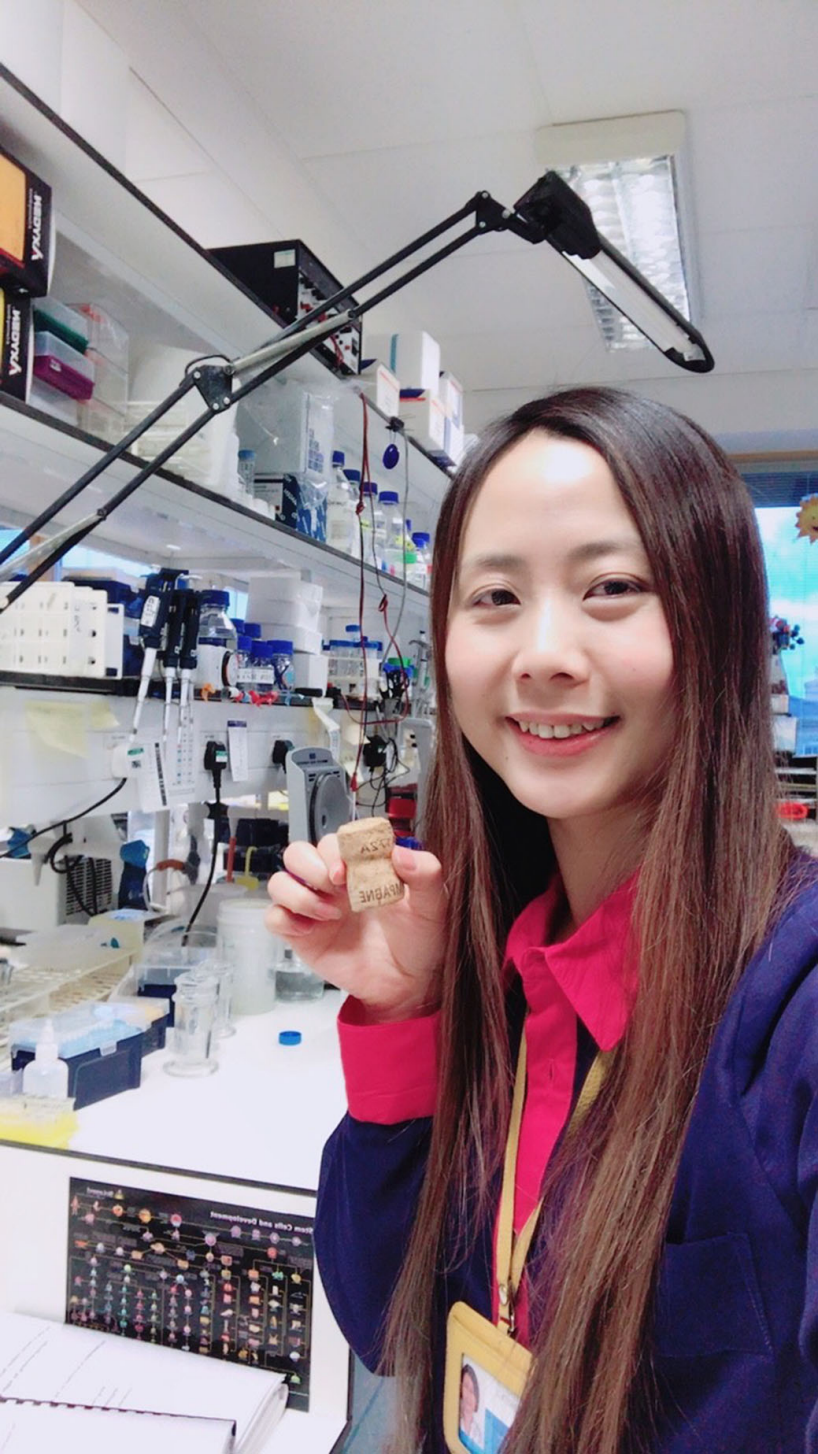


**Ming-Hsuan Wen**

**Describe your scientific journey and your current research focus**

It started with a research project at university as an intern in the labs, and I watched a PhD student show me how to derive umbilical cord stem cells from human tissues. That was the first time I learnt how capable stem cells are, and I was amazed by how they could be applied to many applications. After that, I spent seven years working on a broad range of stem cells for various projects in Taiwan and was awarded a government scholarship to study abroad. That was when I transitioned to study cellular reprogramming under the supervision of Sir John Gurdon. For over nine years, I investigated a fundamental but controversial topic: does nuclear reprogramming require cell division and DNA synthesis? Before the COVID-19 outbreak, I dreamed of being an academic but adjusted my direction afterwards. The world is changing dramatically, and I want to work on tasks that would be applicable and benefit human health. Last year, I seized the chance to translate my research work into commercialisation ideas and founded NUWA Therapeutics to provide state-of-the-art stem cells for novel therapies.

**Who or what inspired you to become a scientist?**

An animated biography I watched about Marie Curie while I was an elementary school student. Her dedication to science inspired me to position myself to become a STEM scientist.

**How would you explain the main finding of your paper?**

In the lifespan of animals, body cells age and malfunction over time. We show that frog oocytes contain critical factors that can revert body cells to its youth and healthy state, and this reversion can be completed in one day.

**What are the potential implications of this finding for your field of research?**

Our finding presents a new method that can turn human body cells into stem cells and provide new stem cell resources for cell therapy. The robust and fast reprogramming process enables the production of autologous stem cells and easy manipulation to design different research models, e.g. organoids and gene-editing cells.

**Figure:**
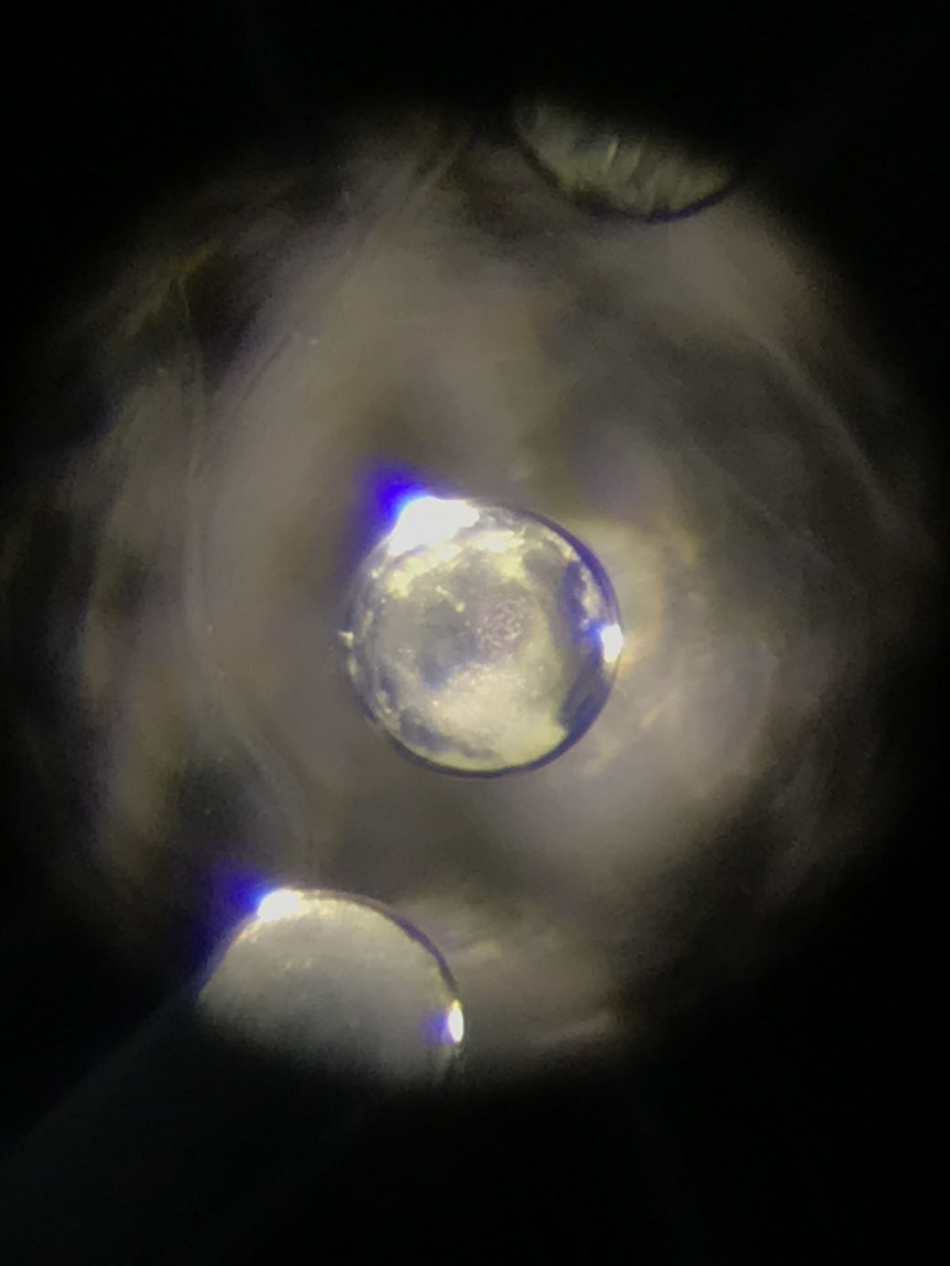
The germinal vesicle of *Xenopus* meiotic oocyte contains the magic factors to reprogramme cells in a day.

**Which part of this research project was the most rewarding?**

The research project becomes the proof of concept of our invention. In the future, we can apply our technology to develop novel therapies and improve the quality of people's lives.

**What do you enjoy most about being an early-career researcher?**

I began my PhD as a pure scientist and become an academic entrepreneur afterwards. Although everything I face is challenging, including the experiments for my project and the vast transition of roles in my life, the most rewarding is that I can always explore different options and enjoy living my life, like stem cells.

**What piece of advice would you give to the next generation of researchers?**

There are always challenges while you are addressing scientific questions. Be honest and be creative. You will learn how to answer questions and find your way to the future.

**What's next for you?**

I want to be an academic entrepreneur, work hard, work smart, and invent valuable technology to benefit the world.

**How do you feel about transitioning from a pure scientist to an academic entrepreneur?**

In the beginning, it was difficult to adapt. My mindset about how I can create value has changed tremendously. Occasionally, I feel dumb or junior along the way, but I am also surprised at how fast I can grow unconsciously and be proud of myself.
